# Ethnopharmacological Use and Biological Activities of *Tragia involucrata* L.

**DOI:** 10.1155/2020/8848676

**Published:** 2020-12-15

**Authors:** Mumtaz S. Pallie, Pathirage K. Perera, Nishantha Kumarasinghe, Menuka Arawwawala, Charitha L. Goonasekara

**Affiliations:** ^1^Faculty of Medicine, General Sir John Kotelawala Defence University, Ratmalana, Sri Lanka; ^2^Institute for Indigenous Medicine, University of Colombo, Colombo, Sri Lanka; ^3^Industrial Technology Institute, Colombo, Sri Lanka

## Abstract

Plants have been utilized as medicines to treat various ailments since ancient times. Formulations made by plant materials have been used in traditional, complementary, and alternative medicine and remain widespread in both developing and developed countries. In developing countries, traditional medicines are widely practiced due to its accessibility and affordability, while in developed countries, complementary and alternative medicine are widely popular due to the adverse effects of chemical drugs. *Tragia involucrata* Linn. (family: Euphorbiaceae) is a highly used medicinal plant used in both Sri Lankan and Indian traditional medical systems. Since this plant is a weed, it is being extensively destroyed due to the lack of knowledge regarding the medicinal value of this plant. Hence, the objective of this study was to collect data on the medicinal value of this plant by correlating its scientifically validated biological activities with its ethnopharmacological uses. An attempt was made to gather as much information available regarding the ethnopharmacological uses and scientifically validated biological activities of *Tragia involucrata* through authentic traditional texts, scientific journals, and other authentic texts regarding medicinal plants. Thus, the review provides an insight to the capability of *Tragia involucrata* to be used as a monoherbal formulation for diseases pertaining to multiple systems of the body. With all the scientifically validated biological activities and the ethnopharmacological uses, *Tragia involucrata* may qualify as a potent candidate to be developed into a phytomedicine to be utilized as both a preventive and as a therapeutic agent.

## 1. Introduction

Plants have been in use as medicines in different formulations to treat various ailments since ancient times. Even at present, medicinal plants play a key role in world health. Use of traditional, complementary, and alternative medicine, which mainly uses plant material for their formulations, remains widespread in both developing and developed countries. According to the World Health Organization (WHO), about 65–80% of the world's population which lives in developing countries depends essentially on medicinal plants for their primary healthcare [1]. Due to this wide use of medicinal plants, the WHO has recommended the initiation of studies to identify and characterize new herbal preparations from traditionally known plants and the development of new effective therapeutic agents, especially in the areas where there is a lack of modern drugs, such as for chronic diseases [2].

*Tragia involucrata* Linn. (family: Euphorbiaceae) is a medicinal plant, which has been used for centuries in Sri Lankan Traditional Medicine as well as in the Ayurveda medical system [3, 4]. This plant is mainly found and used by South Asian countries such as Sri Lanka, India, and Bangladesh. The ethnopharmacological uses of TI illustrates that it has been used in the treatment of disorders in different systems of the body. TI has a great market potential due to its abundant medicinal values and thus has been scientifically investigated for a variety of biological activities.

TI is a weed and therefore propagates easily and survives in harsh weather conditions. Although this plant has been in use for thousands of years, at present, the public is not aware of the medicinal value of this plant, and since it causes a severe stinging effect when touched, the plant is being extensively destroyed especially in Sri Lanka. Due to the destruction of the plant, it is restricted to certain districts of the country. Therefore, the aim of this study was to collect data on the medicinal value of this plant by correlating its biological activities with its ethnopharmacological uses in order to interpret the importance of the plant so that it may be conserved. This review also provides an insight to the capability of *Tragia involucrata* to be used as a monoherbal formulation for noncommunicable diseases due to its many scientifically validated biological activities and its many ethnopharmacological uses which date backs to thousands of years.

## 2. Botanical Evaluation of *Tragia involucrata* Linn

*Tragia involucrata* L. belonging to family Euphorbiaceae is commonly known as Wel Kahambiliya, Helkahambiliya [5], Kahambiliya, or Kasambiliya [6] in Sinhala and Indian stinging nettle or climbing nettle in English. It is called as Indian stinging nettle or climbing nettle in English. In Tamil, the plant is called Ambu or Cherukanjuru [5], and in Sanskrit, it is called Duralabha, Dusparcha, Grahini, and Kachchura [5]. It is a well-known fact that this perennial herb with hispid stem and leaves causes injurious itching and stinging which limit the tangibility. The Sinhala name Kahambiliya is derived from the vesicant effect of TI which causes stinging and itchiness on the skin.

In the book “A revised handbook to the Flora of Ceylon [7],” four types of plants are mentioned under the genus Tragia. They are *Tragia hispida* Willd., *Tragia involucrata* L., *Tragia plukenetii* Radcliffe-Smith, and *Tragia muelleriana* Pax and Hoffm. The vernacular name given for *Tragia hispida* Willd.*, Tragia involucrata* L., and *Tragia plukenetti* Radcliffe-Smith are Wel kahambiliya. A vernacular name had not been given to *Tragia muelleriana* Pax and Hoffm. in Dassanayake and Clayton [7].

### 2.1. Morphology

**Figure 1:**
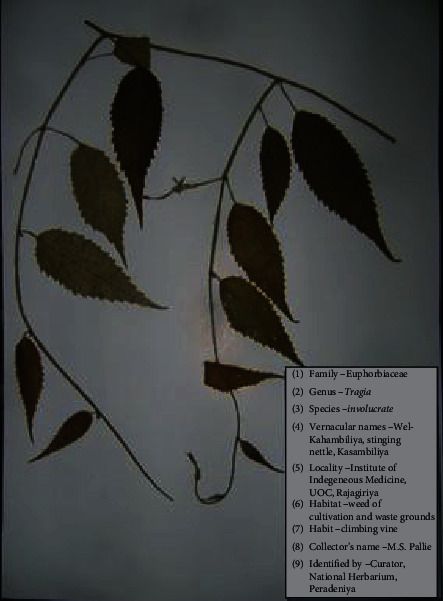
Voucher specimen of *Tragia involucrata*.

TI [5, 7] is a perennial, densely hispid-pubescent herb, with scattered, stinging hairs throughout (Figure 1). The stem is elongate, slender, and twining. Leaves are simple, alternate, serrate, stipulate, 2.5–12.5 cm long, 2–4.5 cm broad, densely hispid-pubescent. Regular, unisexual, and apetalous flowers are borne in terminal axillary. The flowering period is February, March, and June. Fruit is a capsule of 8 mm diameter, 3-lobed, more or less hispid. Seeds are subglobose, grayish brown, and smooth, with slight mottling. TI is geographically distributed in India, Sri Lanka, Burma, and China. In Sri Lanka, it is common in Jaffna, Anuradhapura, Minneriya, Galle, and Matara, as a weed of cultivation and waste grounds.

**Figure 2:**
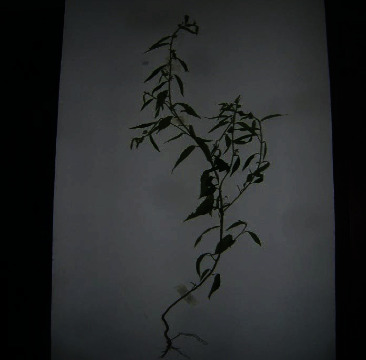
The vine type of *Tragia involucrata*.

**Figure 3:**
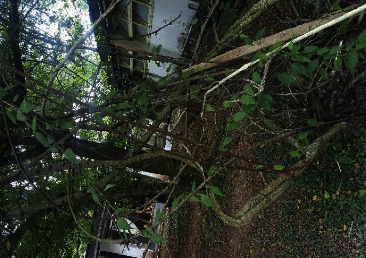
The shrub type of *Tragia involucrata*.

Moreover, there are two morphologically different plants which are known by the vernacular name “Wel Kahambiliya” in Sri Lanka (unpublished data). One plant being a vine (Figure 2) and the other being a shrub (Figure 3). Both plants have been identified by the National Herbarium, Department of National Botanic Gardens, Peradeniya, Sri Lanka, as *Tragia involucrata* L. According to Dassanayake and Clayton [7], the morphological characters which have been used for the “key to the species” in Tragia species under family Euphorbiaceae are the shape of the leaf (whether palmately lobed or simple), base of the leaf (whether cuneate or cordate), and fruiting calyx (whether lobes are linear, variously toothed and stellately spreading, and exposing fruit or broadly ovate, enclosing fruit). The nature of the stem is not considered as a key. Therefore, both the abovementioned two morphological forms fall into the same species. In Dassanayake and Clayton [7], the common stem type for Tragia species has been mentioned as climbing or twining. Hence, it would be worth conducting further investigations into the genetic composition of these two morphologically distinct forms, in order to understand their taxonomical stand in comparison to each other.

### 2.2. Wel Kahambiliya (TI) as a Substitute

As mentioned in Ayurveda Pharmacopoeia, Wel Kahambiliya is used as a substitute for Dhanwayasa, Duralabha, and Yawasa in Sri Lanka [8]. According to most Ayurvedic texts, however, Dhanwayasa and Duralabha are considered one plant, having the botanical name as *Fagonia cretica* Linn. belonging to family Zygophyllaceae [9], and the texts mention that they are synonyms for each other. In Ayurveda authentic text Bhavaprakasha [10], it is mentioned that Duralabha and Yawasa are two different plants. The botanical name of Yawasa is *Alhagi camelorum* Fisch and belongs to family Fabaceae. According to the book, “Medicinal Plants (Indigenous and Exotic) Used in Ceylon [5]” by D.M.A. Jayaweera, the vernacular name of *Alhagi camelorum* is mentioned as Wel Kahambiliya, which further notes that *Alhagi camelorum* is not found in Sri Lanka. Dhanwayasa or Duralabha and Yawasa could be having the same ethnopharmacological properties as *Tragia involucrata*, and thus, the latter is used as a substitute in Sri Lanka due to its availability in the region. The same reason could have led to the use of the vernacular name “Wel Kahambiliya” for those other plants as well.

## 3. Study Design

Data for the study were collected using relevant authentic texts as well as scientific journal articles. Authentic texts that were used for the study were prominent texts used in Ayurveda medicine and Sri Lankan indigenous medicine. The main treatise in Ayurveda which were used in the study included Charaka Samhita [4], Sushruta Samhita [11], Ashtangahrida Samhita [10], and Bhavaprakasha Nighantu [10]. The Sri Lankan texts which were used related to Ayurveda and indigenous medicine were Sarartha Samgraha [3] by King Buddhadasa (340–368 AC) and Ayurveda pharmacopoeia volume II and Ayurveda pharmacopoeia volume III.

**Figure 4:**
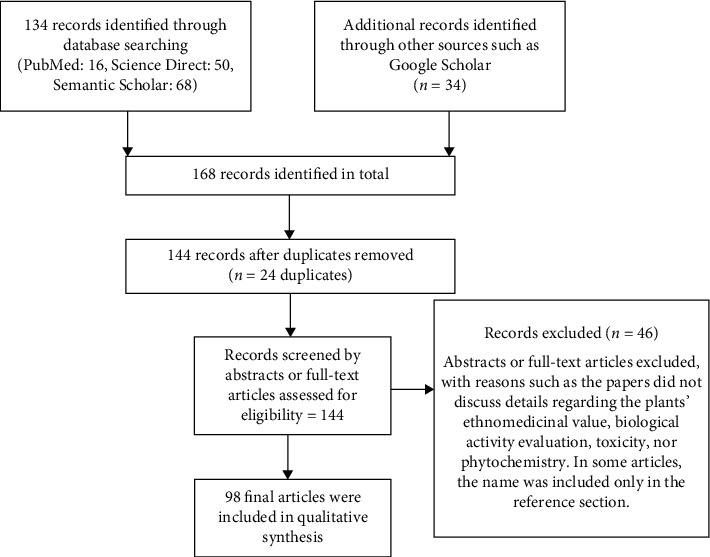
Flow diagram of the study selection process.

Published studies reporting details regarding the biological activities, phytochemistry, and ethnomedicinal uses of *Tragia involucrata* was undertaken according to Figure 4 (illustrates a flow diagram of the study selection process). A comprehensive search of the literature was conducted in the following databases: PubMed (U.S. National Library of Medicine, USA), ScienceDirect (RELX group, Netherlands), and Semantic Scholar (Allen Institute for Artificial Intelligence, USA) for studies published between 1^st^ January 1984 and 31^st^ December 2018. The following medical headings and keywords were used for the search: “*Tragia involucrata*,” “Biological activities of *Tragia involucrata*,” “Phytochemistry of *Tragia involucrata*,” and “Ethnomedicinal use of *Tragia involucrata*.” From a total of 168 results, 24 were excluded because of duplication, 46 being irrelevant judged on the abstract or full paper. Finally, 98 articles were included in this review.

## 4. Ethnomedicinal Uses of *Tragia involucrata* L

The earliest documentation of the ethnopharmacological use of *Tragia involucrata* dates back to 1^st^ century AD. The three main treatises of Ayurveda, Charaka Samhita, Sushruta Samhita, and Vagbhata Samhita, have mentioned TI by the vernacular name of Vrishchakali. In Charaka Samhita [4] documented in 1^st^ century AD, TI is mentioned under Apasmara Chikitsa (treatment for epilepsy). In Sushruta Samhita [11] documented in 4^th^ century AD, TI is mentioned under Jwara Chikitsa (treatment for fever), and in Vagbhata/Ashtangahrida Samhita [10] documented in 5^th^ century AD, TI is mentioned under Chikitsa Sthana as an ingredient of Vidaryadi Gritha, a preparation made from cow's ghee, internally used for disorders of the respiratory tract symptoms.

In Sri Lanka, the earliest documentation of TI goes back to the reign of King Buddhadasa (341–370). The physician king of Sri Lanka compiled “Sarartha Samgraha,” a comprehensive medical treatise, in Sanskrit. In Sarartha Samgraha [3], TI comes under a group of drugs called “welpasmul” (roots of five climbers), comprising of *Ipomoea mauritiana*, *Hemides musindicus, Tragia involucrata*, *Tinospora cordifolia,* and *Pergularia daemia*. The decoction is made from the root of these five climbers mainly for urinary tract disorders.

However, it appears that the use of TI in ethnomedicine has not been for the treatment of a single specific ailment but for a range of unrelated disorders. Various ethnomedicinal uses of TI, as gathered as mentioned in numerous articles and books related to Sri Lankan Traditional Medicine and Ayurveda medicine, are shown in Table 1. The ethnomedicinal use of TI spreads across disorders associated with a range of bodily systems. Therefore, Table 1 demonstrates these ethnomedical uses being categorized according to the body systems, ailments, and the parts of the plant used.

## 5. Biological Activities and Phytochemicals Found in *Tragia involucrata*

A number of controlled researches both in vitro and in vivo have been carried out to scientifically validate the ethnopharmacological properties of TI. Owing to the broader use of TI as an ethnomedicine in traditional medical practice, a larger number of investigations have devoted to scientifically evaluate its biological activities. Many of these studies have supported the value of TI in treating diseases pertaining to the major systems of the body and have further shown to possess a range of biological activities such as antibacterial/microbial activity, antidiabetic/hyperglycaemic activity, antioxidant activity, and anti-inflammatory activity. Since phytochemicals are responsible for these biological activities, simultaneous research carried out on phytochemical analysis of the whole part or parts of TI show that it is rich with phytochemicals, which agrees with its broader biological activities. The major phytochemical groups found to be present in different extracts of *Tragia involucrata*, the whole plant or its parts, as at present, are summarized in Table 2. These phytochemicals are distributed in different parts of the plant and are extractable with different solvent systems. Accordingly, the ethnomedicinal uses of *Tragia involucrata* also varies depending on the part of the plant involved. Further details into the biological activities of *Tragia involucrata*, shown by different parts of the plant under different extraction methods, and the chemical compounds identified as the potential biologically active ingredient are discussed and summarized in Table 3.

### 5.1. Antimicrobial Activity of *Tragia involucrata*

Many studies have been carried out to investigate the antimicrobial activity of *Tragia involucrata* (TI) against a number of microorganisms because of its ethnomedicinal use in wound healing and infections [71, 72]. These antibacterial studies are summarized in Table 4.

The most widely studied part of the plant is the leaf. Few studies have also been investigated for the antimicrobial activity in the stem and the root. The antibacterial activity appears to have depended on the solvent which was used to extract TI rather than the plant part. With respect to the solvents used for the extraction of TI for above antimicrobial studies, it appears that extracts of more polar solvents such as ethyl acetate, acetone, and methanol produced potent antimicrobial activity while that of less polar solvents such as petroleum ether was less active. Aqueous extracts of TI, on the other hand, showed very low antibacterial activity.

Most of the antibacterial investigations had been carried out against Gram-negative bacteria, mostly on *Escherichia coli*, while the choice of Gram-positive bacteria being *Staphylococcus aureus*. All the different extracts of TI leaf, which were tested, have shown potent anti-*S. aureus* activity [78].

Also the wound healing activity, which can be explained by antimicrobial activity, was investigated using the methanolic root extract [69] and Shellsol [79] isolated from fresh TI leaves. The test was carried out on *S. aureus*-induced excision wounds, and the extracts were topically applied. Both the extract and compound were active and showed complete healing of the wound. Furthermore, antibacterial studies with fractionated TI leaf extracts have identified vinyl hexyl ether, Shellsol, and 2-methylnonane as the active compounds possessing antibacterial properties [80, 81]. Moreover, Shellsol isolated from TI leaves seems to be very potent against *S. aureus* Gram-positive bacteria [69, 73]. Another mechanism of action for the antibacterial activity of TI could be through quorum quenching. Bacteria depend on quorum sensing, which is a communication process of bacteria, to regulate gene expression for important cellular processes that are essential for surveillance, survival, and adaptation to their changing environments [82]. Quorum quenching is the inhibition of this quorum sensing. A study has shown that the aqueous leaf extract of TI possess quorum quenching activity [83].

The TI plant showed antifungal activity as well. A study carried out by Panda et al. [70] investigated the ethyl acetate extract of TI root against *Malassezia furfur* fungus. This study showed that the zone of inhibition of EAE of TI root was comparable to that of ketoconazole, which was used as the control. In another study, the ethanol and methanol extract of TI stem was used against *Aspergillus niger* and *Rhizopus arrhizus*, which also gave positive results. In the same study performed by Panda et al. [70], 10, 13-dimethoxy-17-(6-methylheptan-2-yl)-2, 3, 4, 7, 8, 9, 10, 11, 12, 13, 14, 15, 16, 17-tetradecahydro-1H-cyclopenta[a]phenanthrene and 3-(2,4-dimethoxyphenyl)-6,7-dimethoxy-2,3- dihydrochro-men-4-one identified from TI root have shown both antibacterial and antifungal effects. A latest study performed by Gupta et al. [84] showed that the leaf extract of TI possessed antifungal activity against *Chaetomium globosum* and few other pathogenic fungi.

In summary, it is evident that TI possesses antibacterial activity as well as antifungal activity; hence, TI is a potential candidate which qualifies into developing a phytomedicine as an antimicrobial agent.

### 5.2. Anti-Inflammatory Activity of *Tragia involucrata*

Extracts from roots, leaves, and whole plant of TI have been tested to investigate the anti-inflammatory effect. In vivo tests had been carried out on healthy Wistar rats using carrageenan-induced paw oedema and cotton pellet granuloma methods. Different solvent extracts had been used such as aqueous, methanolic, petroleum ether, and chloroform. All the extracts at the tested doses showed positive results both orally as well as intraperitoneal [73, 80, 81, 85–87]. The active component Shellsol has shown positive results for anti-inflammatory activity [73], further indicating that TI potentially mediates its antibacterial as well as anti-inflammatory activity via Shellsol.

### 5.3. Antidiabetic Activity of *Tragia involucrata*

*Tragia involucrata* (TI) has been used for diabetes mellitus in traditional medicine practiced in South Asian countries for centuries. In Sri Lankan traditional medical system, the decoction made out of TI whole plant is used for diabetes mellitus [88]. Using a single plant to prepare decoction is rare in traditional medicine; hence, it depicts the efficacy of TI towards treating diabetes mellitus. Many studies have been performed to investigate the antidiabetic activity of TI, both in vitro and in vivo, and discussed.

In vivo studies have been carried out using diabetes-induced rats. The rats had been induced with alloxan [19, 89] which mimic type I diabetes mellitus and streptozotocin-nicotinamide [12], and high-fat diet and low doses of streptozotocin [90] were also used to mimic type II diabetes mellitus. TI extracts showed potent antidiabetic activity in both types. The hypolipidaemic activity was also investigated during the same antidiabetic study because of the effect of insulin on triglyceride metabolism secondarily causing hyperlipidaemia. TI extracts normalized the values of the lipid profile, which showed that TI possesses insulin mimicking action.

An in vitro antidiabetic study [91] was carried out with the leaf extract of TI using *α*-amylase inhibition assay. The study showed that the extract restrained effective *α*-amylase enzyme inhibitory activity. *α*-Amylase is a protein enzyme which hydrolyses alpha bonds of large, alpha-linked polysaccharides, such as starch and glycogen, yielding glucose and maltose. By inhibiting *α*-amylase enzyme, the breakdown of polysaccharides is hindered, and thereby, the digestion of starch and glycogen is obstructed, and the release of glucose into the blood is inhibited [92]. This is a popular strategy for the treatment of disorders in carbohydrate uptake such as diabetes mellitus and obesity.

To add onto the benefits of TI in diabetes because of its antibacterial activities as discussed previously, TI has also shown potent activity against pathogens which cause diabetic foot ulcers and urinary tract infections [93], which are common complications of diabetes mellitus.

### 5.4. Antioxidant Activity of *Tragia involucrata*

A few antioxidant tests have been carried out using the whole plant and the aerial parts of TI. Alcoholic and ethyl acetate extracts were used for the tests. Most of the studies were in vitro studies using methods such as the free radical scavenging activity (IC 50), ABTS and DPPH radical scavenging methods, Griess reagent method, Phosphomolybdenum method, superoxide dismutase by nitroblue tetrazolium method, and superoxide radical scavenging method. Alcoholic and ethyl acetate extracts of TI showed potent antioxidant activities [51, 89, 94, 95].

### 5.5. Antinociceptive Activity of *Tragia involucrata*

Analgesic activity of TI has been investigated in vivo using acetic acid-induced writing and radian heat analgesiometer methods. Different solvent extracts of the root and the whole plant have been used both orally and intraperitoneally to investigate the activity which proved positive for the tested doses [65, 85, 96].

### 5.6. Antiparasitic Activity of *Tragia involucrata*

Different types of parasites have been used to investigate the antiparasitic activity of TI. Anthelmintic activity was investigated using earthworms and aquarium worms, and the extracts caused paralysis and death of the worms at the tested dose [62, 87]. Larvicidal activity of root and leaf extracts of TI checked using mosquito larvae showed positive results [64, 97]. Also, phagodeterrence, oviposition deterrence, and mosquito repellant activities were also checked in adult female and gravid female mosquitos which showed positive results [58, 97].

### 5.7. Diuretic Activity of *Tragia involucrata*

In Ayurveda and traditional Sri Lankan medicine, TI is used in dysuria and other conditions related to the urinary tract. Hence, diuretic action has been evaluated using different extracts from the root and decoction made from the whole plant. The tests were carried out using healthy Wistar rats. The results showed that the activity of the aqueous root extract and the decoction of the whole plant was most potent as a diuretic, and other extracts such as petroleum ether and chloroform extracts had mild activities [67, 87].

### 5.8. Antitumor Activity of *Tragia involucrata*

Hexane and ethyl acetate extracts of the aerial parts of TI were used on Ehrlich's ascites carcinoma (EAC) bearing mice to investigate the antitumor effect. The extract proved to have antitumor activity at the tested doses [98]. Cytotoxic activity of aerial parts of TI was explored using MTT assay in an in vitro test which also showed potent antitumor activity [99]. Furthermore, the in vitro study performed on Ehrlich's ascites carcinoma-induced albino mice showed anticancer activity in the TI ethyl acetate extract [99].

### 5.9. Other Biological Activities of *Tragia involucrata*

An in vitro study on the antiarthritic activity of the TI leaf extract which has been investigated [100] showed the extracts having potent activity. Yadav et al. [61] shows antihistamine activity of 5-hydroxy-1-methylpiperidin-2-one (5-HMP) isolated from the TI leaf extract, which further found to be mediated through the formation of protein-ligand complex by binding to human serum albumin [61]. Also, a few in vivo studies have been performed to check antifertility activity [51], antiepileptic activity [101, 102], antihistamine activity [60], hepatoprotective activity [103], and nephroprotective activity [104] using different extracts of different parts of TI plant. All these activities showed positive results for the used extract and for the given dose.

## 6. Toxicities Associated with *Tragia involucrata*

TI consists of stinging hair/trichomes with sharp siliceous points which can be found on the whole plant. When the trichome is touched, the tip breaks triggering a basal pump mechanism which acts as a hypodermic syringe and release calcium oxalate and toxic peptides such as Shellsol [105]. These toxins on physical contact cause severe itching, burning pain, and inflammation that may persist for few hours to few days. These proteins like any other proteins are denatured once dried, and therefore, the inflammatory symptoms are present mildly in the dried plant. Furthermore, the water solubility of calcium oxalate is 0.67 mg/L (20°C). Therefore, the plant decoctions which is the medicinal preparation used in traditional medicine does not contain the toxins which cause the inflammatory action.

A number of in vivo investigations have been performed to assess possible toxic effects of various decoctions/extractions prepared of TI, which generally indicated that there is no toxicity associated with TI. These studies had been carried out with the whole plant, aerial parts, and leaf, extracted with various solvents such as water, methanol, petroleum ether, chloroform, and ethyl acetate. Most of the toxicity studies had been assessed up to the 14^th^ day of daily administration of the TI extract using different doses.

Acute toxicity performed using the aqueous extract of whole plant of TI for fourteen days showed negative results at a dose of 5000 mg/kg on healthy male Wistar rats [105]. The methanolic extract of whole plant and leaf did not show 14 days oral acute toxicity at a dose of 2000 mg/kg/day on healthy Wistar rats and Swiss albino mice [12, 101]. The ethyl acetate extract of the aerial part of TI was given to healthy Swiss albino mice through intraperitoneal administration [51] using different doses. Doses of 60, 75, and 90 mg/kg for 14 days IP did not show any toxicity or mortality.

Velu and Malipeddi [100] carried out an in vitro haemolytic activity for the TI leaf extract using human erythrocytes from healthy volunteers. The leaf extract of different solvents such as petroleum ether, chloroform, ethyl acetate, and aqueous extracts showed no haemolytic activity suggesting the nontoxic nature of TI towards erythrocytes.

## 7. Correlation between the Biological Activities and the Ethnomedicinal Uses of *Tragia involucrata*

Ethnomedicinal use of a medicinal plant correlates with the underlining biological activity possessed by the plant. As discussed earlier, *Tragia involucrata* possesses an abundance of ethnomedicinal uses pertaining to different systems of the body, and many of these uses can be correlated to a range of biological activities of the plant that have been scientifically validated by using systematically controlled in vitro and in vivo experiments. In certain instances, the proven biological activity does not correlate directly to the mentioned disease itself but relates to the underlying pathological condition which causes the disease. Furthermore, the medicinal outcome may be due not to the presence of one biological activity but due to a combination of activities.

One such example is TI's ability to act as a febrifuge. This therapeutic indication is found in most of the Ayurveda authentic texts and in Sri Lankan traditional medical texts. According to the traditional medical text Thalapathepiliyam, the whole plant of TI along with 4 other herbs, each comprising 12 g, are added to 1920 ml water to make the decoction of 240 ml, which is consumed daily. Fever can be a symptom of an infection. Therefore, the febrifuge action by TI could be mediated through its anti-inflammatory and antimicrobial activities. Furthermore, one of the psychopharmacological studies performed by Choudhuri Nag et al. [107] using TI methanol fraction of the root extract showed significant central nervous system depressant action, which also includes the decrease of body temperature. As shown by Samy et al. [69], the aqueous leaf extract of TI showed positive results for acute and subacute anti-inflammatory effects at doses of 50, 100, 200, 300, and 400 mg/kg on albino rats. Moreover, in a study carried out by Panda et al. [70], the ethyl acetate extract of TI root at a dose of 250 mg/kg showed a potent antimicrobial effect against many strains of Gram-positive and Gram-negative bacteria and 3 types of fungi. However, the information on the extractable amounts of ingredients in those traditional decoctions are not indicated. Therefore, in terms of the dose administered, the ethnopharmacological data cannot be compared with the effective doses of various biological activities. Moreover, since the method of extractions between these preparations is also different, the compositions and doses of active ingredients can be varied.

Furthermore, the diseases pertaining to the gastrointestinal tract which comes under ethnomedicinal uses such as dysentery [5] and haemorrhoids [24] are due to inflammation and microbial infection. Therefore, the anti-inflammatory [69, 85] and antimicrobial action [70] of TI as mentioned above can be correlated to the fact why it has been used to cure the aforesaid diseases mentioned in the ethnomedicinal uses. The traditional medical text Thalapathepiliyam [108] mentions that 15 g of the root of TI is made into a decoction with three other plants for bloody dysentery. It also mentions that 30 g of TI root, together with the root of another medicinal plant, is made into a decoction at 240 ml for haemorrhoids, in which this decoction is taken orally at a dose of 120 ml each morning and evening.

Ethnomedicinal uses of TI for diseases concerning the respiratory system [19, 20] such as asthma, cough, and bronchitis and integumentary system [19, 30] such as skin diseases are caused by allergies or microbial activity which in turn stimulate inflammation and histamine release. Hence, anti-inflammatory [85], antihistamine [61], and antimicrobial [70] actions can eliminate these diseases. To evaluate the antihistamine effect of TI, Yadav et al. [61] isolated the potent bioactive molecule, 5-hydroxy-1-methylpiperidin-2-one, from the methanol extract of TI leaves. At a dose of 12.5 mg/kg, this compound showed muscle relaxant, bronchodilating, and antiallergic effects, as tested on histamine-induced muscle contraction in the ileum, bronchoconstriction in the bronchioles, and triple response in the skin of guinea pig. To relieve the asthmatic condition, ethnomedically, the root of TI and two other plants, taken in equal portions, is boiled with rice-washed water [108] and used orally.

Some of the ethnomedicinal uses, such as for diabetes, can be directly correlated to scientifically proven biological activities. The antidiabetic activity [17, 18] of TI has been scientifically validated through in vitro and in vivo studies [12, 19, 65, 89, 91]. According to the study performed by Farook and Atlee [12], the oral administration of the aqueous ethanolic extract of TI whole plant showed potent antidiabetic activity on streptozotocin-nicotinamide-induced type 2 diabetes mellitus in rats, at doses 250 and 500 mg/kg. Similarly, in the ethnomedicinal use, 60 g of the dried and pulverized TI whole plant was prepared into a decoction of 240 ml [88] and used at 120 ml each twice a day. The therapeutic human dose of TI in this decoction, calculated by measuring its extractable matter, was 110 mg/kg [67].

Other therapeutic indications, such as the wound healing action, have been carried out by Samy et al. [80], which showed that 50 µg/kg of Shellsol, isolated from TI, exhibited complete healing after 24 days on *Staphylococcus aureus*-induced excision wound in albino rats. In ethnomedicinal use, the aerial parts of TI and two other plants are ground together into a paste and applied on wounds for wound healing [108]. Furthermore, TI is included in a group of drugs called “welpasmul,” meaning the roots of five climbers, which is used for all types of kidney diseases. These herbs are used at a weight of 12 g each, and decoction is made at 240 ml for daily consumption [3]. In agreement, the ethanol leaf extract of TI at dose 250 and 500 mg/kg showed potent nephroprotective activity against acetaminophen-induced toxicity in male albino rats on a study performed by Palani et al. [104]. Its diuretic action was also studied by Pallie et al. [67], which showed dose-dependent diuretic activity of the TI decoction on healthy rats at 550, 1100, 1650, and 2200 mg/kg. The therapeutic rat dose of this TI decoction was 550 mg/kg.

**Figure 5:**
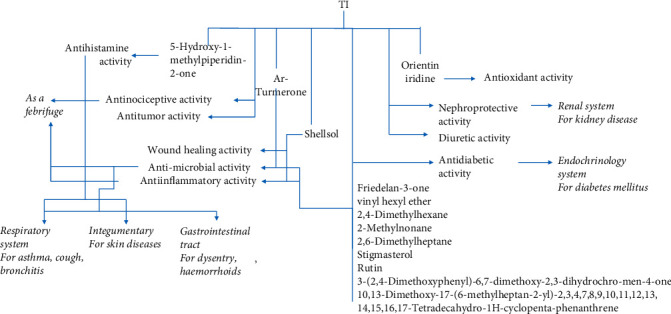
A schematic for the mechanism of action of major ethnopharmacological activities of TI. The validated biological activities of TI have been related to the ethnomedicinal use and the suggested active chemical ingredient.

The mechanism of action/s of ethnopharmacological activities of TI, based on the information currently available and described in the current manuscript, are summarized in Figure 5.

## 8. Discussion

In this study, an attempt was made to analyze available information with on phytochemistry, ethnomedicinal uses, and biological activities of the medicinal herb, *Tragia involucrata*, and to explore the correlation between its ethnomedicinal uses with the related biological activities. This study was therefore aimed to scientifically analyze the potential of *Tragia involucrata* having many therapeutic indications, which appears to be applicable in curing diseases pertaining to most of the body systems. In agreement with the broader nature of the ethnomedicinal use of TI, treating a number of diseases rather than a specific disease, TI has shown to possess a range of different biological activities. Moreover, the fact that TI possesses a variety of biological activities which can interplay to relieve symptoms in a particular ailment makes TI as a remedy with a broader therapeutic value. For example, having activities of antibacterial and analgesic for treating an infection and having activities of antidiabetic, antioxidant, and antibacterial for treating diabetes can be shown. It is currently not clear whether all these activities could be mediated by TI by triggering the activation or inhibition of a biochemical pathway that would be common to all the body systems. It would be interesting, therefore, to investigate the mechanism of action of TI in depth to clearly understand its broader therapeutic indications. Although most of the biological activities have been scientifically proven, except for the antioxidant activity, the mechanism of action of other biological activities have not been scientifically explained in literature. Further research needs to be carried out to investigate the mechanisms of action of these biological activities to get a more clear understanding about the pharmacodynamics of TI.

Regarding the available literature on the phytochemistry of TI, it is clear that only a number of chemical compounds have been isolated so far from TI. Also, most of these chemicals have been studied for their antimicrobial or anti-inflammatory actions alone. These chemicals have not been investigated for multiple biological activities so far. Therefore, studies should also be focused in isolating biologically active chemicals from TI and carry out investigations to understand its mechanism of action responsible for multiple activities.

Furthermore, no studies were found which evaluated the activities of TI in human since all of the studies were either in vitro studies in animals or in vivo studies. Therefore, it should be cautioned when generalizing the conclusions of those studies to the human population, despite the fact that TI has been in use for centuries in traditional medical systems. The way to bridge this gap is through extending those studies to the clinical level to scientifically evaluate the effectiveness of the plant on humans as well as to gain knowledge on its common side effects and drug interactions. Randomized clinical trials are required to be performed as future studies in order to scientifically prove the safety and efficacy of TI to be used as a phytomedicine. It will strengthen the validation of the therapeutic efficacy of this medicinal plant.

## 9. Conclusion

In conclusion, this review was presented in the hope to discover a medicinal herb which has the potential to function in multiple systems of the body and which possess a range of different biological activities. It is of great importance to understand whether *Tragia involucrata* stimulates or inhibits a common biochemical pathway in all the body systems, to trigger those activities. Hence, it would be interesting to investigate the mechanism of action of TI in depth to clearly understand its broader therapeutic indications. Since most of the activities are preclinical trials, it would be necessary to carry out randomized controlled human trials to determine whether TI can be developed into a phytomedicine to be used as both as a preventive and a therapeutic agent.

## Figures and Tables

**Table 1:** Ethnomedicinal uses of *Tragia involucrata*.

System of the body/main action	Plant part used	Ailment	Reference
(1) Endocrinology system	Whole plant	(i) Madumeha (diabetes mellitus)	[12–18]
	Root	(ii) A major constituent of anti-diabetic formulations	[19]

(2) Digestive system	Whole plant	(i) Appetizer	Ayurveda pharmacopoeia [20]
		(ii) Gastropathy and antiemetic	[17, 21]
		(iii) Used as a mouth wash to cleanse oral cavity	[22]
	Root	(i) Diarrhoea, vomiting, and dysentery	[4]
		(ii) Constipation	[23]
		(iii) Haemorrhoids and gastropathy	[24]
		(iv) Anthelmintic–to get rid of Guinea worms	[11]
	Leaves and roots	(i) Bloody dysentery and stomachache	[4]
		(ii) Constipation, haemorrhoids, and vomiting	[25]

(3) Respiratory system	Whole plant	(i) Asthma	[19, 26]
		(ii) Cough and asthma	Ayurveda pharmacopoeia [20]
		(iii) Bronchitis, pneumonia, and laryngitis	[5]
	Root	(i) Bronchitis	Ayurveda pharmacopoeia [20, 27, 28]
		(ii) Dry cough	[5]
		(iii) Infants acute breathing complications	[29]

(4) Integumentary system	Whole plant	(i) Chronic inflammatory skin diseases (psoriasis, eczema, and seborrheic dermatitis)	[30, 31]
		(ii) Pruritic eruptions	[11]
		(iii) Elephant's skin diseases	[22]
	Root	(i) Pruritic skin eruptions	[24]
		(ii) Skin diseases including leprosy	Ayurveda pharmacopoeia [20, 32]
		(iii) Wounds	Ayurveda pharmacopoeia [20, 32]
		(iv) Abscess	[34]
	Leaves	(i) Skin infection, swellings, children scabies, and eczema in children	[19, 35]
	Fruit	(i) Bloody eruptions	Ayurveda pharmacopoeia [20]
		(ii) Indraluptha (hair loss causing patches of balding/alopecia areata)	Ayurveda pharmacopoeia [4, 20]
	Stem	(i) Dermatitis	[36]
	Leaves and root	(i) Skin diseases	[32]
	Stem and leaves	(i) Skin diseases	[4]

(5) Urinary system	Whole plant	(i) Diuretic	Ayurveda pharmacopoeia [20]
	Root	(i) Diuretic	Ayurveda pharmacopoeia [20]
		(ii) For all urinary problems	[37]
		(iii) Dysuria	[3, 38]
	Stem and leaves	(i) For renal stones	[39]

(6) Cardiovascular system	Whole plant	(i) Cardiotonic	[22]
	Root	(ii) As a blood purifier	[24]

(7) Nervous system	Whole plant	(i) Headache	[40]
	Root	(i) Epilepsy	[4, 41, 42]
		(ii) Headache	Ayurveda pharmacopoeia [20]
		(iii) Migraine	[19]
		(iv) Pain in the limbs	[4]
		(v) Pain in the waist	[34]
		(vi) Melalgia and brachialgia	[24]
	Leaves and roots	(i) Headache, vertigo, and giddiness	[25]
	Fruit	(i) For headaches	[16, 43]

(8) Immune system			
Inflammation	Root	(i) Antiperiodic and depurative	[24]
		(ii) Tumor and elephantiasis	[19, 44]
		(iii) Arthritis and rheumatoid arthritis	[35, 45]
	Leaves	(i) Local swelling of hands and feet	[46]
Fever	Whole plant	(i) For pain and cold extremities due to high fever	Ayurveda pharmacopoeia [20]
		(ii) All types of fever	[37]
		(iii) For typhoid fever	[5]
		(iv) Malarial fever	[47]
	Root	(i) Diaphoretic	[24]
		(ii) Febrifuge	Ayurveda pharmacopoeia [20, 48]
Venereal diseases	Whole plant	(i) Chronic syphilis	Ayurveda pharmacopoeia [20]
	Root	(i) Venereal diseases	[24]
Allergies	Root	(i) Itches due to allergies	[23]

(9) Reproductive system	Whole plant	(i) As an aphrodisiac	[49]
	Stem and leaves	(i) A contraceptive drug called “shanti bori” is made from aerial parts of TI	[50, 51]

(10) Snake and scorpion bites	Whole plant	(i) Scorpion stings and snake bites	[48, 49]
	Roots	(i) Snake bites	[52–55]
	Leaves	(i) For scorpion stings, insect bites, and snake bites	[19, 32]

(11) Miscellaneous	Whole plant	(i) As anticancer agent	[56, 57]
		(ii) As a nutritional source	[49]
		(iii) Mosquito repellent	[58]
		(iv) Ophthalmic diseases	[47]
		(v) Eliminates toxins from the body, energy booster	[22]
	Root	(i) Protection of a new born baby	[34]
		(ii) To warm the body	[36]
		(iii) Ankle sprains and fractures	[59]
	Leaves	(i) Insect repellents	[22]

**Table 2:** Major phytochemicals found in various extracts of different parts of *Tragia involucrata*.

Plant part	Extract	Phytochemical constituent											Reference
		Alkaloids	Coumarins	Catechins	Flavonoids	Glycosides	Phenols	Saponins	Sterols	Steroids	Tannins	Terpenoids	

Leaf	Fresh	+	NT	NT	+	NT	+	+	NT	+	+	+	[60]
	MeOH	+^*∗*^	NT	NT	+^*∗∗*^	+^*∗∗*^	NT	NT	+^*∗∗*^	NT	+^*∗∗*^	NT	^*∗*^[61], ^*∗∗*^[62]
	EtOH	+	NT	−	+	+	−	−	+	+	−	+	[63]
	Hexane	+	NT	−	+	+	−	−	NT	+	−	+	[64]
Root	H_2_O	+^*∗*^	NT	NT	+^*∗∗*^	−^*∗∗*^	NT	+^*∗*^	−^*∗∗*^	−^*∗∗*^	+^*∗∗*^	−^*∗∗*^	^*∗*^[65], ^*∗∗*^[66]
	MeOH	−^*∗∗*^	NT	NT	+^*∗∗*^	−^*∗∗*^	NT	+^*∗∗*^	−^*∗∗*^	−^*∗∗*^	+^*∗∗*^	−^*∗∗*^	^*∗∗*^[66]
	PE	−^*∗∗*^	NT	NT	+^*∗*^	−^*∗∗*^	NT	−^*∗∗*^	+^*∗∗*^	−^*∗∗*^	+^*∗*^	−^*∗∗*^	^*∗*^[65], ^*∗∗*^[66]
	EA	+^*∗∗*^	NT	NT	−^*∗∗*^	−^*∗∗*^	NT	−^*∗∗*^	−^*∗∗*^	−^*∗∗*^	−^*∗∗*^	−^*∗∗*^	^*∗∗*^[66]
	CHL	+^*∗*^	NT	NT	−^*∗∗*^	−^*∗∗*^	NT	−^*∗∗*^	+^*∗∗*^	−^*∗∗*^	−^*∗∗*^	−^*∗∗*^	^*∗*^[65], ^*∗∗*^[66]
Whole plant	Hot water	−	+	NT	+	+	NT	+	+	−	+	+	[67]
	Aq/EtOH	+	NT	NT	+	NT	+	+	+	NT	+	+	[12]
	Aq/MeOH	NT	NT	NT	+	NT	NT	NT	NT	NT	NT	NT	[68]

NT, not tested; +, present; −, negative; MeOH, methanol; EtOH, ethanol; H_2_O/Aq, water; PE, petroleum ether; EA, ethyl acetate; CHL, chloroform. ^*∗*^,^*∗∗*^Relevant references.

**Table 3:** Biologically active phytochemicals isolated from *Tragia involucrata*.

	Phytochemical	Structure	Biological activity	Reference
1	Ar-turmerone (identified by GC-MS)	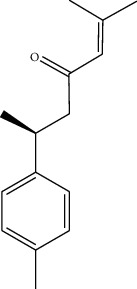	Antimicrobial Wound healing	[17]
2	Friedelan-3-one (identified by GC-MS)	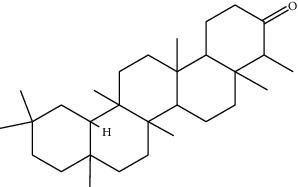	Antimicrobial Anti-inflammatory	[17]
3	Vinyl hexyl ether (identified by GC-MS)	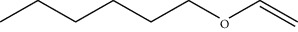	Antimicrobial Anti-inflammatory	[69]
4	Shellsol (identified by GC-MS)	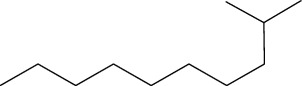	Antimicrobial Anti-inflammatory Wound healing	[69]
5	2,4-Dimethylhexane (identified by GC-MS)	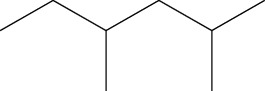	Antimicrobial Anti-inflammatory	[69]
6	2-Methylnonane (identified by GC-MS)	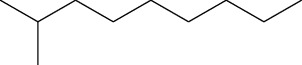	Antimicrobial Anti-inflammatory	[69]
7	2,6-Dimethylheptane (identified by GC-MS)	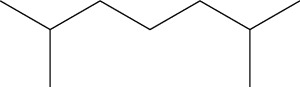	Antimicrobial Anti-inflammatory	[69]
8	Iridine (identified by LC/MS)	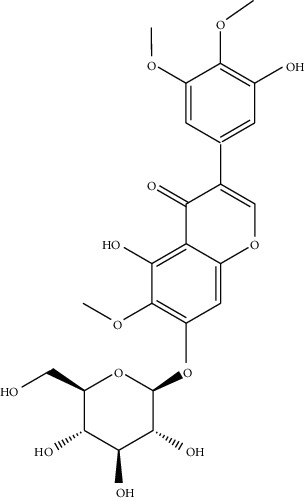	Antioxidant	[68]
9	Orientin (identified by LC/MS)	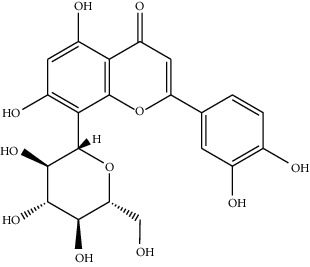	Antioxidant	[68]
10	10,13-Dimethoxy-17-(6-methylheptan-2-yl)-2,3,4,7,8,9,10,11,12,13,14,15,16,17-tetradecahydro-1H-cyclopenta-phenanthrene (identified by IR and H-NMR spectroscopy)	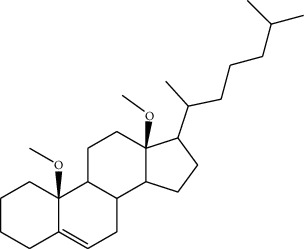	Antimicrobial Anti-inflammatory	[70]
11	Stigmasterol (identified by IR and H-NMR spectroscopy)	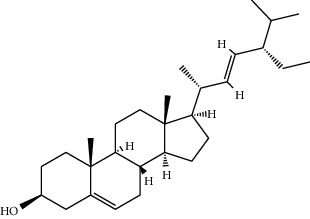	Antimicrobial Anti-inflammatory	[70]
12	Quercetin (identified by IR and H-NMR spectroscopy)	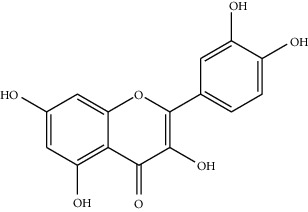	Antimicrobial Anti-inflammatory	[70]
13	Rutin (identified by IR and H-NMR spectroscopy)	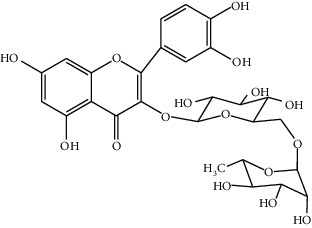	Antimicrobial Anti-inflammatory	[70]
14	3-(2,4-Dimethoxyphenyl)-6,7-dimethoxy-2,3-dihydrochro-men-4-one (identified by IR and H-NMR spectroscopy)	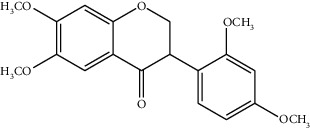	Antimicrobial Anti-inflammatory	[70]

**Table 4:** Summarized findings of antibacterial studies carried out on *Tragia involucrata*.

Type of extract	Bacterial type										Reference
		Gram (+)	Gram (−)									

S.A.	V.C.	P.M.	P.A.	V.D.	K.P.	B.P.	E.C.	P.V.	S.M.
Isolated compounds from TI leaves	+	NT	NT	NT	NT	NT	NT	+	+	NT	[69]
Ethyl acetate extract of root	+	NT	NT	NT	NT	NT	NT	+	NT	NT	[70]
Methanolic leaf extract	+	NT	NT	−	+	+^*∗*^	+	NT	NT	NT	[73],^*∗*^[74]
Acetone leaf extract	NT	NT	NT	NT	NT	NT	NT	+	NT	NT	[60]
Acetone root extract	−	NT	NT	+	NT	+	NT	+	NT	+	[75]
Acetone and methanol leaf extract	+	NT	−	−	NT	−	NT	+	NT	+	[76]
Ethanol leaf extract	+	NT	+	−	NT	−	NT	+	NT	+	[76]
Chloroform stem and ethanol leaf extract	NT	NT	+	+	NT	NT	NT	NT	NT	NT	[63]
Silver nanoparticles synthesized from TI stem	+	NT	NT	NT	NT	NT	NT	+	NT	NT	[77]

S.A., *Staphylococcus aureus*; V.C., *Vibrio cholera*; P.M., *Proteus mirabilis*; P.A., *Pseudomonas aeruginosa*; V.D., *Vibrio damsel*; K.P., *Klebsiella pneumonia*; B.P., *Burkholderia pseudomallei*; E.C., *Escherichia coli*; P.V., *Proteus vulgaris*; S.M., *Serratia marcescens*. NT, not tested. (+), activity present. (−), activity absent. ^*∗*^,^*∗∗*^Relevant references.
